# A review of human cornea finite element modeling: geometry modeling, constitutive modeling, and outlooks

**DOI:** 10.3389/fbioe.2024.1455027

**Published:** 2024-10-15

**Authors:** Guobao Pang, Chenyan Wang, Xiaojun Wang, Xiaona Li, Qiaoyu Meng

**Affiliations:** ^1^ Shanxi Bethune Hospital, Shanxi Academy of Medical Sciences, Tongji Shanxi Hospital, Third Hospital of Shanxi Medical University, Taiyuan, China; ^2^ College of Biomedical Engineering, Taiyuan University of Technology, Taiyuan, China; ^3^ College of Mechanical and Vehicle Engineering, Taiyuan University of Technology, Taiyuan, China; ^4^ Institute of Advanced Structure Technology, Beijing Institute of Technology, Beijing, China

**Keywords:** human cornea, finite element analysis, geometric model, constitutive model, biomechanics property

## Abstract

The cornea is a vital tissue of the human body. The health status of the cornea has a great impact on the quality life of person. There has been a great deal of research on the human cornea biomechancis. However, the difficulty in obtaining the human cornea has greatly limited the research of cornea biomechancis. Using finite element modelling has become a very effective and economical means for studying mechanical properties of human cornea. In this review, the geometrical and constitutive models of the cornea are summarised and analysed, respectively. Some factors affecting of the finite element calculation are discussed. In addition, prospects and challenges for the finite element model of the human cornea are presented. This review will be helpful to researchers performing studies in the relevant fields of human cornea finite element analysis.

## 1 Introduction

**FIGURE 1:**
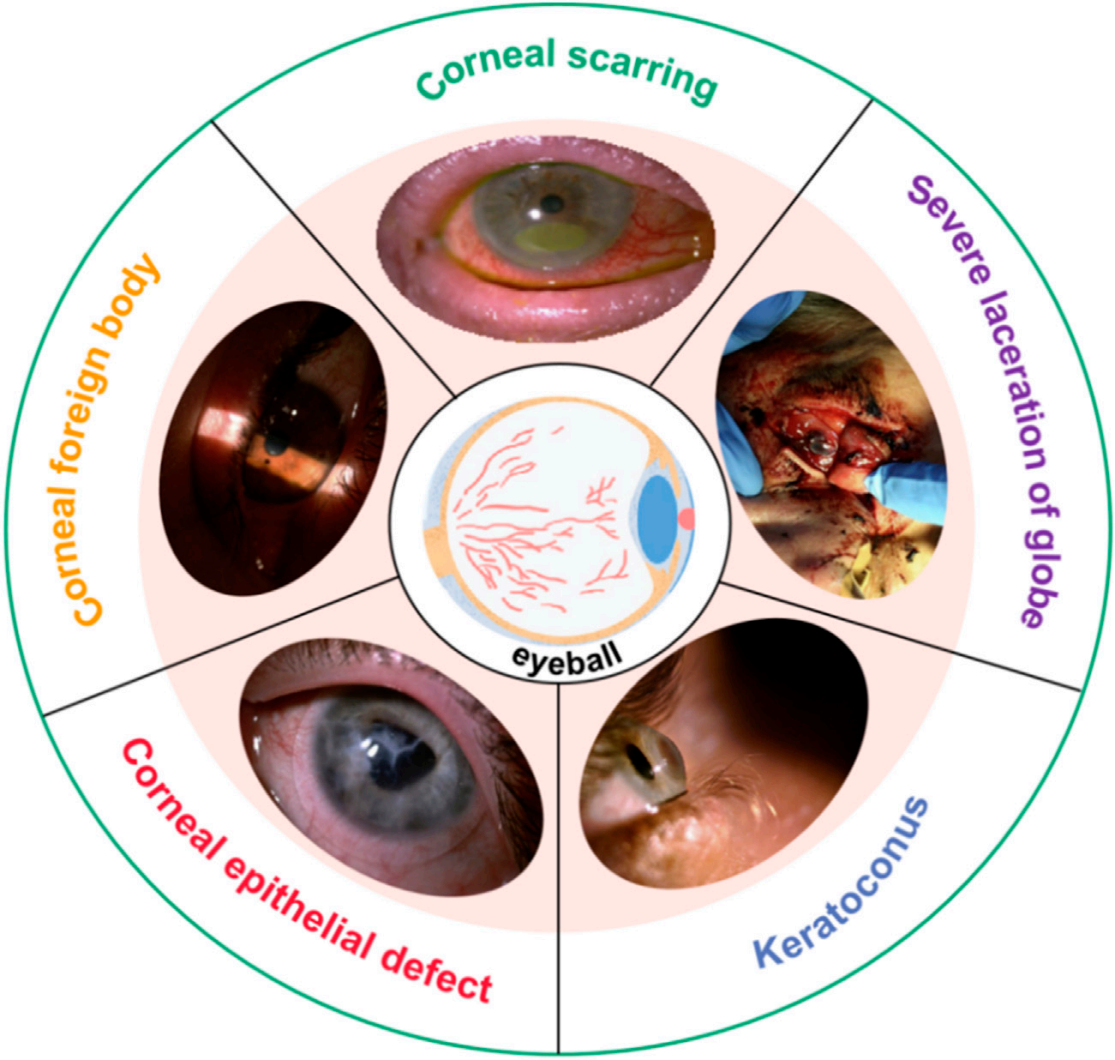
Photos of corneal injury (Barrientez et al., 2019).

The eyes are vital organ of the human. It contains many tissues and delicate structures, such as the cornea, sclera, lens, iris, vitreous, aqueous humor, ciliary muscle, retina, choroid, optic nerve, and aqueous humor (Clevenger et al., 2024; Arestova et al., 2024; Murugan and Cheng, 2022; Neuhuber and Schrödl, 2011; Stefánsson, 2009; Mark, 2010; Fisher, 1977; Jonas et al., 1999). Any slight injury in the eye’s tissues can seriously affect eye function. The cornea is located on the outer surface of the eyeball, and the cornea is also one of the most vulnerable parts of the eyeball. Common corneal diseases include refractive errors (myopia, hyperopia, astigmatism), keratoconus, corneal dystrophy, tumors, inflammation, and blindness (Pinazo-Durán et al., 2016; BITAR et al., 2018; Dutta et al., 2021; Rampat et al., 2021). Figure 1 shows some photos of corneal injury: corneal scarring, severe laceration of the cornea, keratoconus, corneal epithelial defect, and corneal foreign body (Barrientez et al., 2019). For refractive errors, refractive surgery, such as laser *in situ* keratomileusis (LASIK), femtosecond laser-assisted *in situ* keratomileusis (Fs-LASIK), and small-incision lenticule extraction (SMILE), is an effective method (Schiefer et al., 2016; Melki and Azar, 2001; Zhang et al., 2016; Sekundo et al., 2011). Ultraviolet light cross-linking therapy surgery is used to treat keratoconus and corneal dystrophy (Santhiago and Randleman, 2021; Wernli et al., 2013; Santodomingo-Rubido et al., 2022). Severe keratoconus, corneal dilation, and corneal tumors can cause corneal perforation or even blindness. Corneal transplantat surgery will become a kind of important means for the repair vision. These surgeries are very complex and require extensive clinical experience. It also presents a significant challenge for surgeons.

The Finite element analysis (FEA) method is widely used in engineering analysis and calculation, and also widely used in biomechanics field (Nikishkov, 2004; Logg, 2007). Common finite element analysis software include Ansys, Abaqus, Comsol, Adina, Dyna, and Marc. In the field of biomechanics, the appearance of finite element soft provides great convenience for the study of the mechanical properties of human tissues *in vivo*. Such as blood vessels, hearts, bone tissue, cells, and eyeballs, finite element analysis software is often applied (Clough, 1990). Compared with laboratory experiments, computer-based simulation has many advantages. Firstly, finite element analysis can effectively simulate, calculate, analyze and predict various mechanical behaviors of human tissues (Tandale et al., 2022). Secondly, it greatly reduces the experiment cost, especially for the study of human tissues that are difficult to obtain. Thirdly, FEA can avoid human ethical problem (Civalek et al., 2020). Finally, finite element analysis can evaluate the experimental results more quickly, efficiently, and accurately. In the past, for many surgeries or corneal studies, researchers often needed to conduct animal experiments to do relevant research. However, animal corneas are quite different from human corneal tissues, and the results are difficult to directly apply to human eyes. Finite element can give full play to its advantages and facilitate researchers to model and analyze human corneas. For the quantitative assessment of corneal refractive surgery or corneal trauma, the finite element methof has played a great role. In addition, for the study of corneal orthokeratology, finite element analysis can also effectively analyze the biomechanical response of the orthokeratology to the cornea. By establishing LASIK and SMILE surgical models, Wang et al. (2022) quantitatively analyzed the stress distribution and displacement distribution of the corneal surface after two kinds of surgery. She believed that SMILE refractive surgery is superior to LASIK surgery in terms of corneal biomechanical stability. Bao et al. (2018) used finite element modeling to simulate LASIK refractive surgery. The effects of LASIK surgery on corneal biomechanical behavior were analyzed and the results were validated using clinical datasets. The results show that the quantitative analysis of the shape and refractive power was reliable and effective.

Finite element analysis also has some disadvantages. The first is that the geometry of the model is often difficult to exactly correspond to the real shape. In finite element modeling, it is often necessary to simplify the geometry of the model. Different degrees of simplification have different effects on the calculation results. Secondly, the constitutive relationship of biological soft tissue materials is very complex, but in order to facilitate the description of material properties, the finite element method often simplifies the description of the constitutive relationship of materials studied. The effect of the simplification of the constitutive relation on the calculation results can not be ignored. Third, the result of finite element calculation is not a real solution, but a numerical solution. The finite element method divides the computational domain into finite elements by the basic principle of variational and the method of weighted margin (Wang et al., 2021). Select the basis function for each unit, replace the solution of each unit with the linear combination of the basis function. The solution of all the basis function can replace the solution of the whole solution region. As the calculation accuracy becomes higher and higher, the solution will become closer and closer to the real solution. Therefore, for the finite element analysis of human cornea, the accuracy of describing the geometric shape and constitutive relationship of corneal tissue is crucial to the accuracy of the research results.

The finite element technique has greatly promoted the research of cornea-related diseases. Many scholars have proposed that finite element models can accurately predict biomechanical behavior, and it have been proven to have good predictability. Wang et al. (2016) combined FEA and magnetic resonance imaging to estimate the strain on the optic nerve head during horizontal eye movement. Liu et al. (2015) used FEA to study the mechanism of lens capsule rupture. Wu et al. (2021) studied the corneal reshaping mechanism of corneal molding lenses using a numerical model. Bao et al. (2018) analyzed the effects of LASIK on corneal biomechanics using FEA. FEA has been used extensively to study the biomechanical properties of the cornea and related diseases. Generally, the finite element model of the cornea is mainly composed of geometric model and constitutive model. This article summarizes the current research status of the finite element model for the human cornea. The geometric and constitutive model of the human cornea are introduced in Section 2 and Section 3, respectively. Finally, the prospects and outlooks of human cornea finite element model are presented and discussed. The purpose of this article is to provide references and ideas for research on finite element models of the human cornea and provide a framework for future research.

## 2 Corneal geometry model

The sense organ of the visual perception system is called the eye, which is composed of the eyeball and the accessory organ of the eye. The eyeball is nearly spherical in shape and is used to receive light stimulation. The accessory organ of the eye is to protect the eyeball from damage. The eyeball structure is the main part of the optometer (Bekerman et al., 2014; Hong et al., 2023). The normal eyeball has a anteroposity-posterior diameter of about 22.0 mm–24.0 mm, a horizontal diameter of about 23.0 mm, a volume of about 6.5 mL, and a weight of about 7.0 g. The walls and contents of the eyeball together make up the eyeball. The role of the ocular wall is to maintain the shape of the eyeball, including the retina, cornea, choroid, sclera, ciliary body and so on (Alcântara et al., 2023). The transparent cornea and sclera form the outer wall of the eyeball. The contents of the eyeball consist of aqueous humor, vitreous body and lens, which are the pathways for light to enter the eye (Zelentsova et al., 2020; Hammer et al., 2024; Toffoletto et al., 2020). These structures together with the cornea constitute the refractive system of the eyeball. When the external light is focused at the center of the retina after the dioptric system, it is called orthotropia. Nearsightedness and farsightedness are due to the focus of the light to the front and back of the retina respectively. When the light focuses on multiple focal points of different planes on the retina, it is called astigmatism (Holladay et al., 2022). Aqueous humor metabolizes waste in the ocular tissues, maintains intraocular pressure and provides nutrients. If the return of aqueous humor is abnormal, it may cause increased intraocular pressure, which is clinically called glaucoma. The intraocular pressure of normal people ranges from 10 mmHg to 21 mmHg (1 mmHg = 0.133 kPa). 98% of vitreous body is water, which mainly plays a role in metabolizing products secreted by tissues and alleviating the influence of vibration on surrounding tissues. The lens is elastic and transparent, equivalent to a convex lens of 19 D (Da Silva and Lira, 2022). The ciliary muscle regulates the lens so that objects can be clearly imaged in the retina. With the age, the elasticity of the lens, and the adjustment ability of the ciliary muscle gradually weaken, resulting in presbyopia. Cataracts are formed when the lens is damaged and cloudy. There are many contents of the eyeball and its functions are complex, and any abnormality in any part may affect vision and even affect the function of other systems in the body (Stepp and Menko, 2021; Buffault et al., 2020; Sun et al., 2020).

As the outer barrier protects the eyes, the cornea is required to withstand intraocular pressure and the fluid motion from eye movement. The cornea is the first interface that light encounters as it enters the eye, and is transparent with a circular shape (Meek and Knupp, 2015). The center 1/3 of the anterior surface of the cornea is known as the optical zone and has a shape similar to that of a spherical surface (Gardner, 2015). The horizontal and vertical diameter of the human cornea is about 11.00 mm–12.00 mm, and the radius of curvature of the anterior and posterior surfaces is about 7.8 mm and 6.8 mm, respectively (Meek and Knupp, 2015; Gardner, 2015). The width of the human cornea is approximately 1.5 mm–2.0 mm at its edge. Researchers often use the mean value of corneal geometric data to build an ideal corneal model. However, the cornea contributes roughly 70% of the total refractive power of the eye (Meek and Knupp, 2015). Even very small changes in the corneal curvature and thickness can have a significant impact on its refractive power. Therefore, there will be differences between the ideal geometric model and the patient-specific in the simulation and calculation results. In this section, the ideal geometric model and the patient-specific geometric model are summarized. The layered geometric models of the cornea are also reviewed.

### 2.1 Ideal geometric model

#### 2.1.1 Two-dimensional model

**FIGURE 2:**
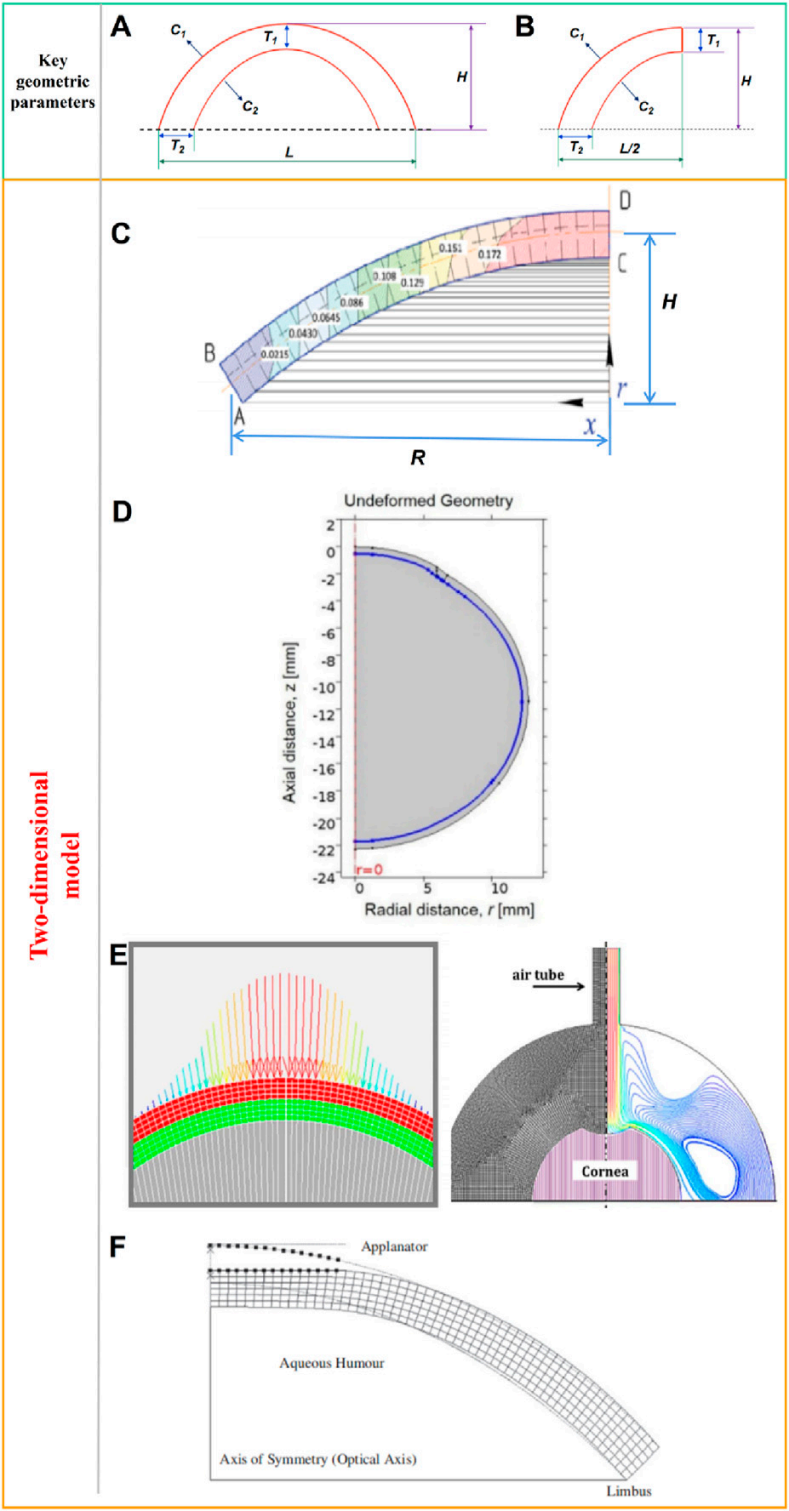
**(A)** Ideal two-dimensional axisymmetric geometric model and key geometric parameters, **(B)** Half of a two-dimensional axisymmetric geometric model, **(C)** two-dimensional corneal model, **(D)** two-dimensional axisymmetric whole-eye geometry model, **(E)** two-dimensional axisymmetric model with limbus, **(F)** two-dimensional axisymmetric model (Spevak and Babailov, 2020; Nguyen et al., 2019; Kling et al., 2014; Kwon et al., 2008).

The cornea can be regarded as hemispherical or semi-ellipsoid shape. Many researchers have built models based on the mean curvature and thickness of the cornea to simplify the calculation. As shown in Figure 2A, some key geometric parameters for the cornea model including the corneal center thickness *T*_1_, the corneal margin thickness *T*_2_, the anterior radius of curvature *C*_1_, the posterior radius of curvature *C*_2_, the corneal height *H*, and the corneal diameter *L*. Due to the axisymmetric shape of the ideal model, many researchers choose half of the model for calculation to reduce the operation cost, as shown in Figure 2B. There are many ways to obtain these key parameters of cornea model (*T*_*1*_, *T*_*2*_, *C*_*1*_, *C*_*2*_, *H*, and *L*). Experimental measurements, nondestructive detection techniques, and literature reported are effective ways to obtain these key parameters. Each of these methods has its advantages and disadvantages. For experimental measurements, due to the advanced detection technology was not developed in the past, researchers often could only obtain geometric data of the human cornea by measuring the cornea *in vitro*. Many corneas are harvested from cadaver corneas or surgical scraps. Corneal data measured by *in vitro* experiments have some shortcomings. First, a large number of corneal cells die after a short period of time (Wilson, 2020; Méthot et al., 2020; Ruan et al., 2021). The time from corneal acquisition to laboratory measurement often causes corneal cell death and even dramatic changes in corneal geometry. Secondly, corneal transport often needs to be preserved in solution, which will also cause distortion of the true shape of the cornea. Moreover, the number of human corneas available is extremely small, resulting in the scarcity of valid experimental data for reference. Therefore, there are many defects in the method of obtaining the geometric data of human cornea from *in vitro* corneal experiment, and the data accuracy maybe not very accurate. Although *in vitro* measurements of corneal data have a variety of shortcomings. Cornea acquisition is not easy, *in vitro* acquisition data is also an important reference for our research. Hjortdal et al. (Hjortdal and Peter, 1995) studied human eyes taken from *postmortem* bodies. Time of death was less than 96 h. The eyeballs were frozen and stored at −25°C. The central corneal thickness was measured by an optical thickness gauge. The slit light illumines the center of the cornea at an angle of 38.5°, while the viewing angle between the camera and the central surface of the cornea is 0°. The central corneal thickness was 569 μm. A corneal contour image in a meridian was obtained by a computer prism device, and 3 points on the cornea were selected to estimate the central curvature radius of the cornea. Repeated measurements were made and the average corneal curvature was estimated to be 7,998 μm. These *in vitro* measurements are close to those obtained by nondestructive testing. The values of *L* and *H* were not measured in this study. For nondestructive detection techniques, at present, the commonly used detection devices are X-ray computed tomography (CT), Magnetic Resonance Imaging (MRI), Corneal visualization Scheimpflug technology (Corvis ST), and three-dimensional anterior segment analyzer (Pentacam and Orbscan II) (Withers et al., 2021; Zwanenburg et al., 2021; Weiskopf et al., 2021; Salouti et al., 2020; Jo et al., 2023; Askarian et al., 2022). CT now enables high-precision three-dimensional inspections of the internal structure. The principle of CT is to reconstruct the image through the X-ray 3D scanning data. Especially for complex objects such as eyeballs, CT technology has a unique advantage, its measurement accuracy and repeatability are high. Similar to CT technology, MRI technology has higher detection accuracy. But MRI technology has the advantage of sparing the body from X-rays. The corneal visualization Scheimpflug technology (Corvis ST) is a common detection device used in the clinic. The detection principle of Corvis ST is to apply a pulse air stream to the cornea *in vivo*. The detection system uses a high-speed imager to dynamically record the deformation and shape reduction of the cornea under the action of the air stream in real time. The system can also measure central corneal thickness (CCT), biomechanical corrected intraocular pressure (IOP), pulse air pressure, corneal vertex displacement change with time, rebound rate, corneal deformation amplitude, first and second flattening time, corneal arc length change crest distance, first and second flattening length, corneal curvature change and other data. Corvis ST plays an important role in the diagnosis of glaucoma, keratoconus and preoperative screening of refractive surgery, and is currently a highly respected biomechanical detection device. Pentacam and Orbscan II are the common three dimensional corneal anterior segment analyzers. Pentacam instrument has high accuracy and repeatability, wide measurement coverage, comprehensive description of corneal morphological characteristics, high resolution, fast detection process and no damage, and is more sensitive to the diagnosis of eye disease than Orbscan II, which is currently the most commonly used and effective detection instrument for the diagnosis of eye diseaes. The principle is to obtain the image of the front segment through the full Angle rotation Scheimpflug optical principle. The analyzer uses the blue aurora diode to complete the 180° rotation scan within 2 s. Each shooting generates a picture composed of 500 height data, which can automatically generate three-dimensional color stereoscopic images with strong and intuitive image visibility. The system comes with two cameras that can automatically track and correct eye movements, measure the surface shape data, curvature data, thickness, anterior chamber, lens and other anterior segment parameters of the cornea. For the literature, the data in the literature are often obtained through the previous experimental data, empirical data or the average result of some data. These data are often subject to large discrepancies and errors. In conclusion, it is more accurate and easy to obtain these key parameters of cornea model (*T*_*1*_, *T*_*2*_, *C*_*1*_, *C*_*2*_, *H*) by means of nondestructive testing techniques. Among them, Corvis ST and Pentacam have unique advantages in the ease of inspection operation, inspection cost, and detection accuracy. Therefore, Corvis ST and Pentacam are also the most popular detection methods in human cornea research at present.

Spevak and Babailov (2020) established a two-dimensional corneal model as shown in Figure 2C, and analyzed the stress-strain state of the cornea. Spevak and Babailov (2020) made 4 simplifications that reduced the dimension of the problem and reduced the amount of finite element calculation. The first one is that the shape is axisymmetric, without eccentricity; The second is that the cornea is simplified to single layer; The third is the cornea material is elastic and isotropic; The last is the model is under isothermal conditions. According to the data reported in the literature, the shape of the model is set to an ideal curve. The geometric parameters were set as the average values reported in the literature. The curvature of the anterior and posterior surfaces is consistent. Corneal thickness was set at 520 μm; the corneal deflection along the symmetry axis (*H*) was set at 2.5 cm; and the radius along center line (*R*) was set at 5 cm. Nguyen et al. (2019) established a two-dimensional axisymmetric whole-eye geometry model to simulate the Corvis ST detection process through COMSOL. The geometric data of the model are derived from literature reports. The whole golbe diameter was 24 mm. The axisymmetric geometry of eye include cornea, sclera, and vitreous. The model surrounded by the air region. Central corneal thickness was set to 500 μm, anterior radius of curvature and posterior radius of curvature to 8.00 mm and 6.8 mm, respectively. Corneal diameter was 11 mm, as shown in Figure 2D. For sclera area, the thickness at equator was 400 μm; the thickness at posterior pole was 1,000 μm; the radious of curvature was 12 mm.These parameter values all from revelant literature. Kling et al. (2014) established a two-dimensional axisymmetric model to simulate the effect of air column on corneal deformation, as shown in Figure 2E. The corneal curvature and CCT were form the Scheimpflug cross-sectional images. The central thickness of the cornea was 558 μm, the anterior and posterior surface curvature radii were 8.03 mm and 6.86 mm, respectively, and the corneal diameter was 10 mm. The model also takes into account the sclera and limbus. The scleral diameter was 19.5 mm. Kwon et al. (2008) established a simplified two-dimensional axisymmetric finite element model of the human eye, as shown in Figure 2F. The Figure 2F shows the initial state of the cornea and the shape of the cornea after applanated. A series of Goldmann applanation tonometer simulations were performed on the model to investigate the effects of corneal geometry and material properties on IOP readings. The geometry of the anterior and posterior surfaces of the cornea in the model is represented by the following Formula 1:

$$r^{2}+\left(1+Q\right)z^{2}-2zR=0$$ (1)

where the origin is on the (anterior or posterior) surface of the cornea at the optical axis, the *z*-axis points inwards along the optical axis, and *r* is the radial distance from the optical axis. *Q* is an asphericity parameter and *R* is the radius of curvature at the apex. These geometry data from literature. The central thickness of the cornea was 550 μm. The *R* and *Q* of the anterior surface are 7.77 mm and −0.18, respectively. The *R* and *Q* of the posterior surface are 6.40 mm and −0.60, respectively. Here we summarize some values of *T*_1_, *T*_2_, *C*_1_, *C*_2_, *H*, and *L* reported in the literature as shown in Table 1. For a fixed value of corneal curvature, only one of the *L* and *H* values is needed to determine the two dimensional shape. Therefore, the *L* is not indicated in Fernandez et al. (2004), and the *H* is not indicated in Larry et al. (2007), Kling et al. (2014), and Nguyen et al. (2019).

**TABLE 1:** Corneal geometric parameters in the two-dimensional model.

Parameters	Ref.
*C*_1_ = *C*_2_ = 7.56 mm, *T*_1_ = 0.52 mm, *T*_2_ = 0.67 mm, *H* = 2.5 mm	Fernandez et al. (2004)
*T*_1_ = *T*_2_ = 0.52 mm, *L* = 10 mm, *H* = 2.5 mm	Spevak and Babailov (2020)
*C*_1_ = 7.77 mm, *C*_2_ = 6.40 mm, *T*_1_ = *T*_2_ = 0.55 mm, *L* and *H* are from geometry equation	Kwon et al. (2008)
*C*_1_ = *C*_2_ = 7.80 mm, *T*_1_ = 0.61 mm, *T*_2_ = 0.812 mm, *L* = 12.36 mm	Larry et al. (2007)
*C*_1_ = 8.03 mm, *C*_2_ = 6.86 mm, *T*_1_ = *T*_2_ = 0.56 mm, *L* = 10 mm	Kling et al. (2014)
*C*_1_ = 8.0 mm, *C*_2_ = 6.80 mm, *T*_1_ = *T*_2_ = 0.50 mm, *L* = 11 mm	Nguyen et al. (2019)
*C*_1_ = 7.96 mm, *C*_2_ = 6.20 mm, *T*_1_ = 0.549 mm, *T*_2_ = 0.75 mm, *L* = 11.76 mm, *H* = 2.4 mm	Huang et al. (2020)
*C*_1_ = *C*_2_ = 7.97 mm, *L* = 11.49 mm, *H* = 2.5 mm, *T*_1_ and *T*_2_ are based on patient thickness	Meng et al. (2020)

These models have in common that they are two-dimensional symmetric models, and all require the value of *C*, *T*, *H* and *L* for modeling. The advantage of two-dimensional symmetric model is that it reduces the computational cost to a large extent. First of all, compared with the three-dimensional symmetric model, the number of finite element elements of the two-dimensional symmetric model is significantly reduced, which greatly saves the calculation cost. Secondly, due to the symmetry of the model, the physical response of the cornea is symmetrical when it is stressed or deformed. It is often possible to select half of the computational model to analyze the results.

The accuracy of the calculation is determined by the complexity of the geometric model of cornea. However, there are limbus and other tissues around the cornea, and the biomechanical response of the cornea will be affected by the tissues around the cornea when the cornea is stressed. Both Figures 2D,E took into account the cornea limbus, and the calculation results of the model were more accurate than those of Figure 2C. The disavantage of the two-dimensional symmetric models are not negligible either. When the analysis objective is only to observe the changes of corneal cross-section or corneal contour, it is acceptable to simplify the model using two-dimensional structure. When it is necessary to analyze the stress-strain distribution on the surface or inside of the cornea, the two-dimensional model is not sufficient. For example, when simulating corneal trauma or surgery, the stress state and deformation of the cornea at different locations vary greatly. At this case, the two-dimensional model can not meet the research needs. What’s more, the race, the age, the corneal refractive status, and the other multiple factors can lead to the difference of corneal geometric parameters in different people. The average value of the key parameters will cause the error of the model and the error of the calculation result. In addition, the cornea is not completely symmetrical shape, so it is not very reasonable to idealize the cornea as a symmetrical structure and only calculate half of the model.

#### 2.1.2 Three-dimensional model

**FIGURE 3:**
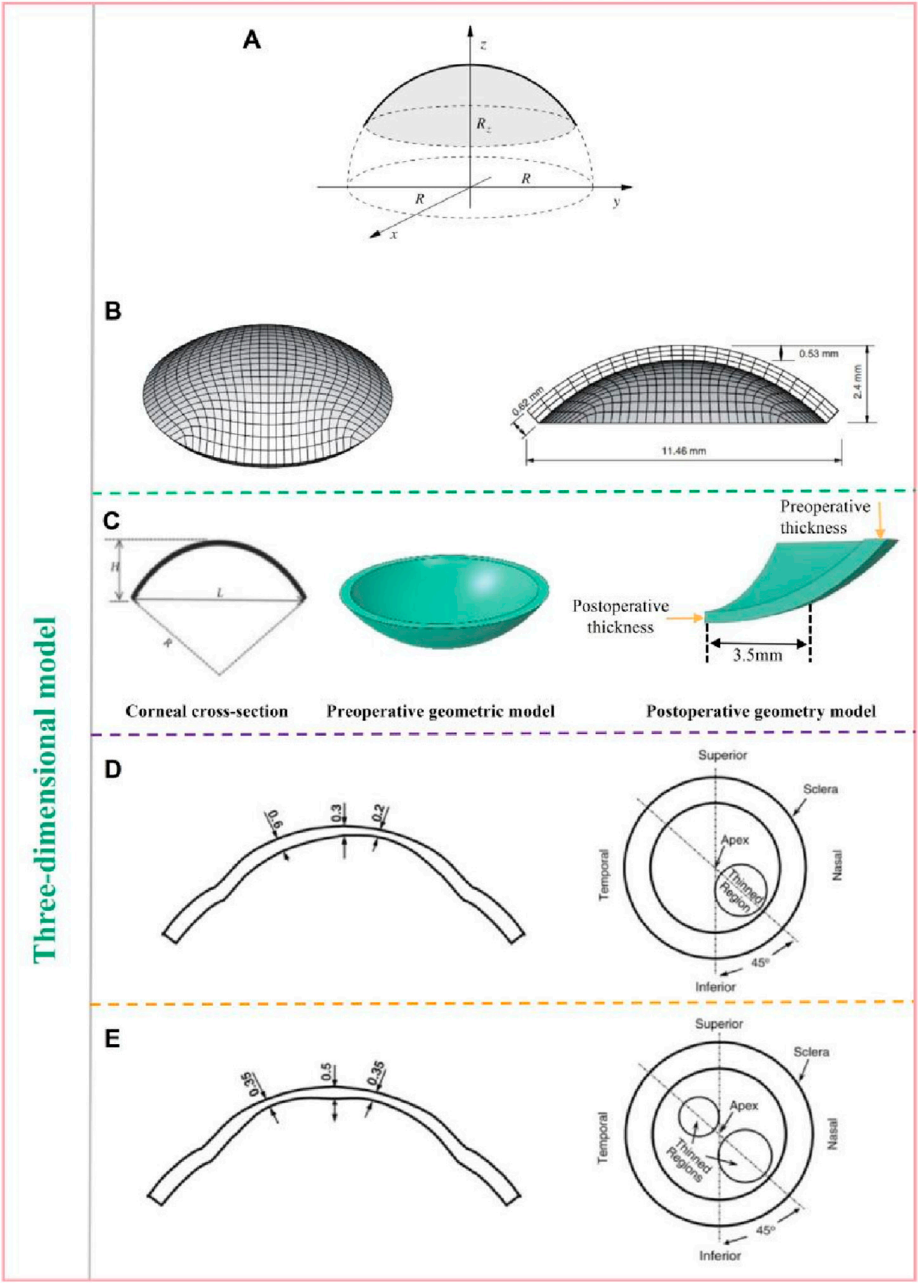
Ideal geometric model: three-dimensional model (Meng et al., 2020; Pandolfi and Manganiello, 2006; Gefen et al., 2009). **(A)** the diagram of ellipsoid. **(B)** the model of cornea and the peripheral region. **(C)** the preoperative and preoperativecorneal model. **(D)** the keratoconus model with one thinning region. **(E)** the keratoconus model with two thinning region.

For some cases where the surface stress distribution or displacement distribution of the cornea is analyzed, the two-dimensional model cannot meet the requirements. The three-dimensional corneal model can observe the changes of the cornea in the three-dimensional space. Pandolfi and Manganiello (2006) regarded the cornea as a long ellipsoid (Figure 3A), used the ellipsoid to approximate the surface of the myopic cornea and astigmatic cornea in cylindrical coordinate system. The equation of ellipsoid is given by Formula 2.

$$\left(1-e^{2}\right)z^{2}+x^{2}+y^{2}=R^{2},e=\sqrt{1-\frac{R^{2}}{R_{Z}^{2}}}$$ (2)

Where *e* is eccentricity, *R* is the maximum radius in the *x* and *y* directions, *R*_z_ is the maximum radius in the *z* direction. The outer in plane diameter of the cornea is 11.46 mm. The maximum elevation at the apex is 2.4 mm. The shell thickness is about 0.53 mm at the center and 0.62 mm at the limbus. The ellipsoid eccentricity *e* is 0.60 for the internal surface and 0.43 for the external surface. The maximum external and internal curvatures are 7.8 and 6.8 mm, respectively. The slope of the cross section at the limbus is 48°. The model are shown in Figure 3B. Meng et al. (2020) selected 34 myopia patients (total 67 eyes) who underwent LASIK in Shanxi Eye Hospital (China), including 15 males (30 eyes) and 19 females (37 eyes). All patients underwent Corvis ST examination before and after surgery. Biomechanical parameters such as CCT and IOP were obtained before and after surgery. The three-dimensional corneal models of 67 patients before and after surgery were established. The changes of corneal collagen stiffness before and after surgery were analyzed. All preoperative and postoperative corneal models were treated as spherical structures, and unified anterior surface curvature radius *R*, height *H* and transverse diameter *L* were used. As shown in Figure 3C, *R* was 7.9 mm, *H* was 2.5 mm, and *L* was 11.5 mm. These values come from data reported by Pandolfi and Holzapfel (2008). The preoperative corneal model used uniform thickness, that is, the thickness of the cornea was the same at different locations. The corresponding preoperative model was established according to the preoperative CCT value of each patient. The difference between preoperative corneal CCT value and postoperative corneal CCT value is considered as the quantity of surgical cutting. According to the quantity of surgical cutting, the cutting was carried out within the 3.5 mm radius of the apex center of the preoperative model to obtain the postoperative model. Figure 3C also shows a schematic of the post-operative quarter corneal model. The difference in the corneal models of these 67 cases was only reflected in the difference in central corneal thickness, and other geometric parameters were not considered in model. For the more complex keratoconus, some researchers have analyzed the biomechanical properties of keratoconus by idealized models. Gefen et al. (2009) established an idealized keratoconus with one and two thinning regions respectively, and analyzed and predicted the changes of refractive and surface stress in keratoconus under different intraocular pressure. The geometric data is derived from published experimental data. Geometric data of key positions were selected for modeling. The maximum and thinnest thickness of the cornea with one thinning region model was set at 0.6 mm and 0.2 mm, respectively, as shown in Figure 3D. The maximum and minimum thicknesses for two thinning regions model are set to 0.5 mm and 0.35 mm, respectively, as shown in Figure 3E.

### 2.2 Patient-specific model

The geometry of the cornea varies from person to person. For a certain cornea, the curvature and thickness of each position on the front and back surfaces of the cornea are also different. The difference in surface curvature and thickness between the front and back will directly lead to the difference in the stress state of the cornea at each position. Therefore, surface curvature and thickness are most obviously different parameters for building patient-specific models.

#### 2.2.1 Two-dimensional model

**FIGURE 4:**
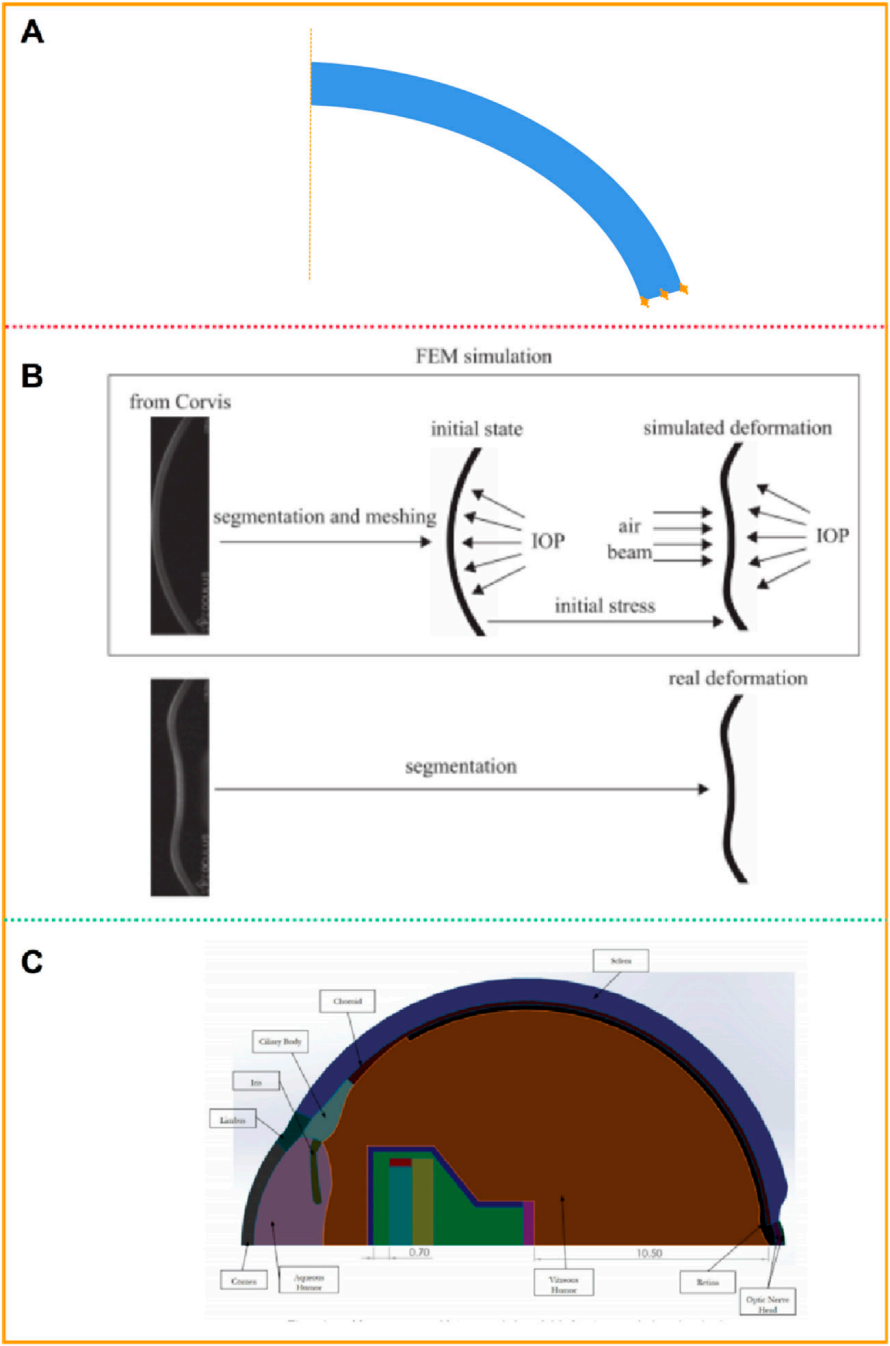
Patient-specific geometric model: two-dimensional model (Jannesari et al., 2018; Lago et al., 2015a; Stark et al., 2021). **(A, B)** two-dimensional geometric model. **(C)** two-dimensional model including cornea, sclera, and other tissues.

The models of normal, myopic or keratoconus cornea based on the anatomical data have a certain gap with the actual corneal morphology. They cannot accurately restore the irregular surface and curvature of the cornea, nor can they accurately predict the changes in the cornea after force. Jannesari et al. (2018) obtained the structure of the cornea without deformation from the images captured by Corvis ST. Using image processing technology, the coordinates of 576 points on the front surface and 576 points on the back surface of the cornea were obtained, and the two-dimensional geometric model of the patient was obtained by interconnecting splines, as shown in Figure 4A. Similarly, Lago et al. (Lago et al., 2015a) used images captured by Corvis ST to build a 2-dimensional corneal model, as shown in Figure 4B. Modeling and simulation were carried out on the images of 12 patients. The corneal thickness distribution in these models ranged from 499 μm to 613 μm, and the intraocular pressure ranged from 8 mmHg to 14.5 mmHg. The deformation of the human cornea during non-contact pressure measurement was simulated, and the properties of corneal materials were calculated by inverse finite element method and genetic algorithm respectively. Stark et al. (Stark et al., 2021) established a two-dimensional model including cornea, sclera, choroid, iris and other tissues, as shown in Figure 4C. The size of the eye was 24.79 mm along anterior-posterior direction and 24 mm in transverse and vertical directions. The anterior and posterior corneal radius of curvature are 7.76 mm and 6.52 mm, respectively. The central corneal thickness is 0.55 mm. The anterior chamber depth is 3.06 mm. The vitreous chamber depth is 16.6 mm. The lens diameter is about 9.03 mm. The limbal anterior and central thickness are 0.704 mm and 0.767 mm, respectively. The limbal posterior and scleral equatorial thickness are 0.728 mm and 0.556 mm, respectively. The posterior pole thickness is 0.834 mm. Data from the U.S Army and its in-house laboratory simulated the analysis of ocular deformation of the cornea under the action of the intraocular projector.

#### 2.2.2 Three-dimensional model

**FIGURE 5:**
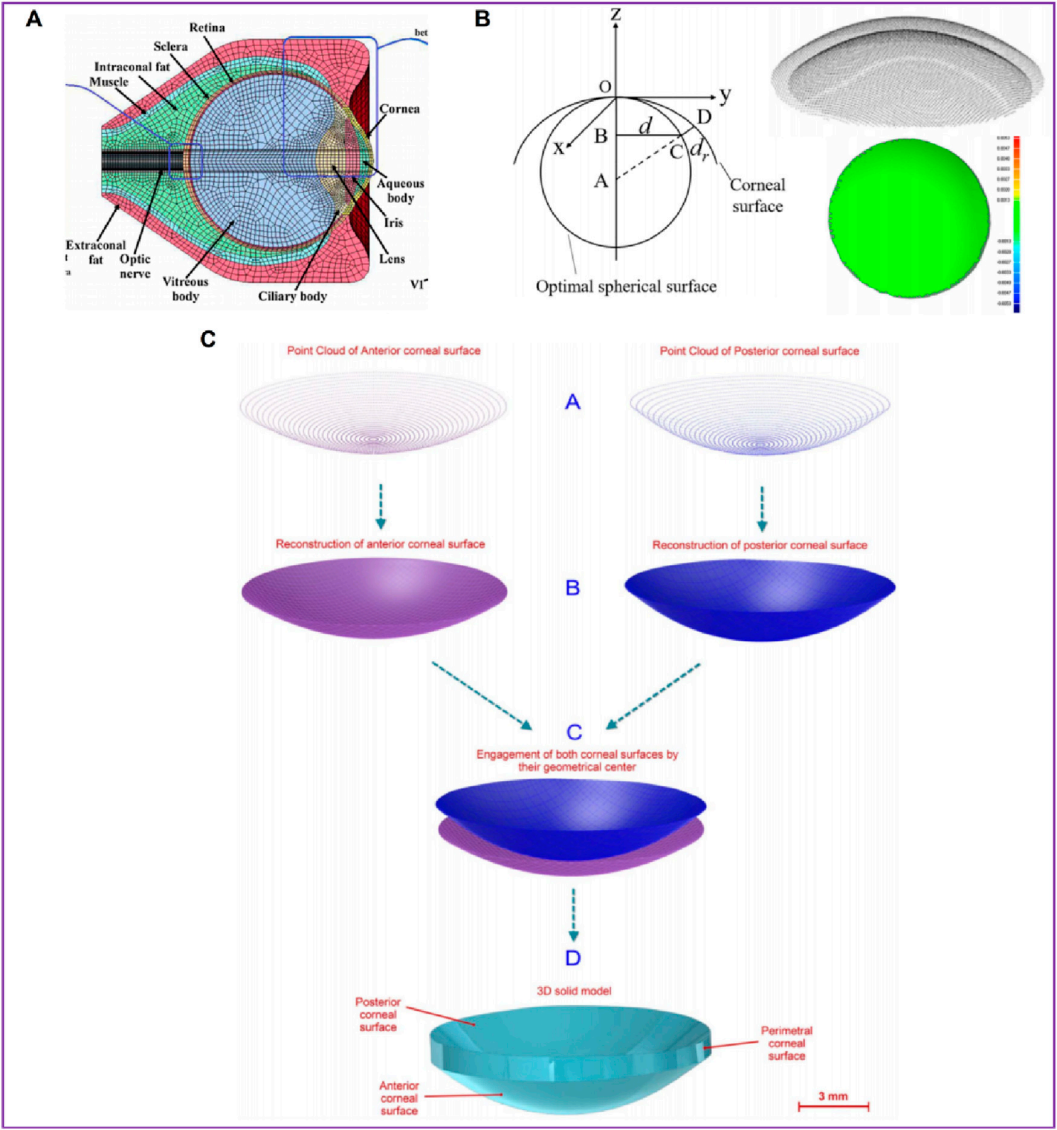
Patient-specific geometric model: three-dimensional model (Wang et al., 2022; Karimi et al., 2018; Cavas et al., 2014). **(A)** the cornea data from CT and MRI. **(B)** the cornea data from Pentacam. **(C)** the cornea data from Sirius corneal topographer.

In order to describe corneal morphology more accurately, many researchers have tried to establish corneal models with the help of detection instruments to study corneal biomechanical properties more accurately. Among them, OCT, CT, MRI, Pentacam and various types of corneal topography instruments are widely used. The basic principles of these instruments are similar, and all of them can accurately describe the morphological characteristics of the cornea and restore the surface morphology of the cornea. Karimi et al. (2018) collected patients’ eye data through CT and MRI, and established eye models including cornea, sclera, optic nerve, extraocular muscle and other tissues (Figure 5A). Wang et al. (2022) collected the radial distance between each point on the corneal surface and the optimal spherical surface within the range of 8 mm × 8 mm, the corresponding thickness value of each point on the front surface of the cornea, and the optimal spherical radius value by Pentacam topographic instrument. A three-dimensional space rectangular coordinate system was established, the coordinate values of all points on the cornea in the *z*-axis direction were obtained, and the coordinate point data of the front and back surface of the cornea were imported into the reverse modeling software Geomagic to form the front and back surface profile of the cornea (Figure 5B). The models after LASIK surgery and SMILE surgery were established to accurately analyze the biomechanical changes of the cornea caused by the two kinds of surgery. Roy et al. (Roy and Dupps, 2011) used a three-dimensional anterior segment analyzer combined Zernike polynomial and Gaussian polynomial to fit the geometric expression of cornea. Cavas et al. (2014) used Sirius corneal topographer to obtain point cloud fitting of the geometric shapes of the front and back surfaces of the cornea to produce corneal curves. The modeling process is shown in Figure 5C. Especially for some irregular corneas, such as keratoconus, the thickness is uneven and asymmetrical, the apex position of the cone and the steepening area are not fixed, the surface curvature changes greatly, and the geometric shape is more irregular than that of normal corneas, with great individual differences. It is very effective to obtain corneal geometry by various scanning instruments, and finite element models can be built more accurately. Lago et al. (2015b) reconstructed the patient’s keratoconus model. The keratoconus images and topographical data were collected by Pentacam. The restoration of corneal curvature in keratoconus patients after corneal ring segment implantation was simulated.

### 2.3 Layered geometric models

**FIGURE 6:**
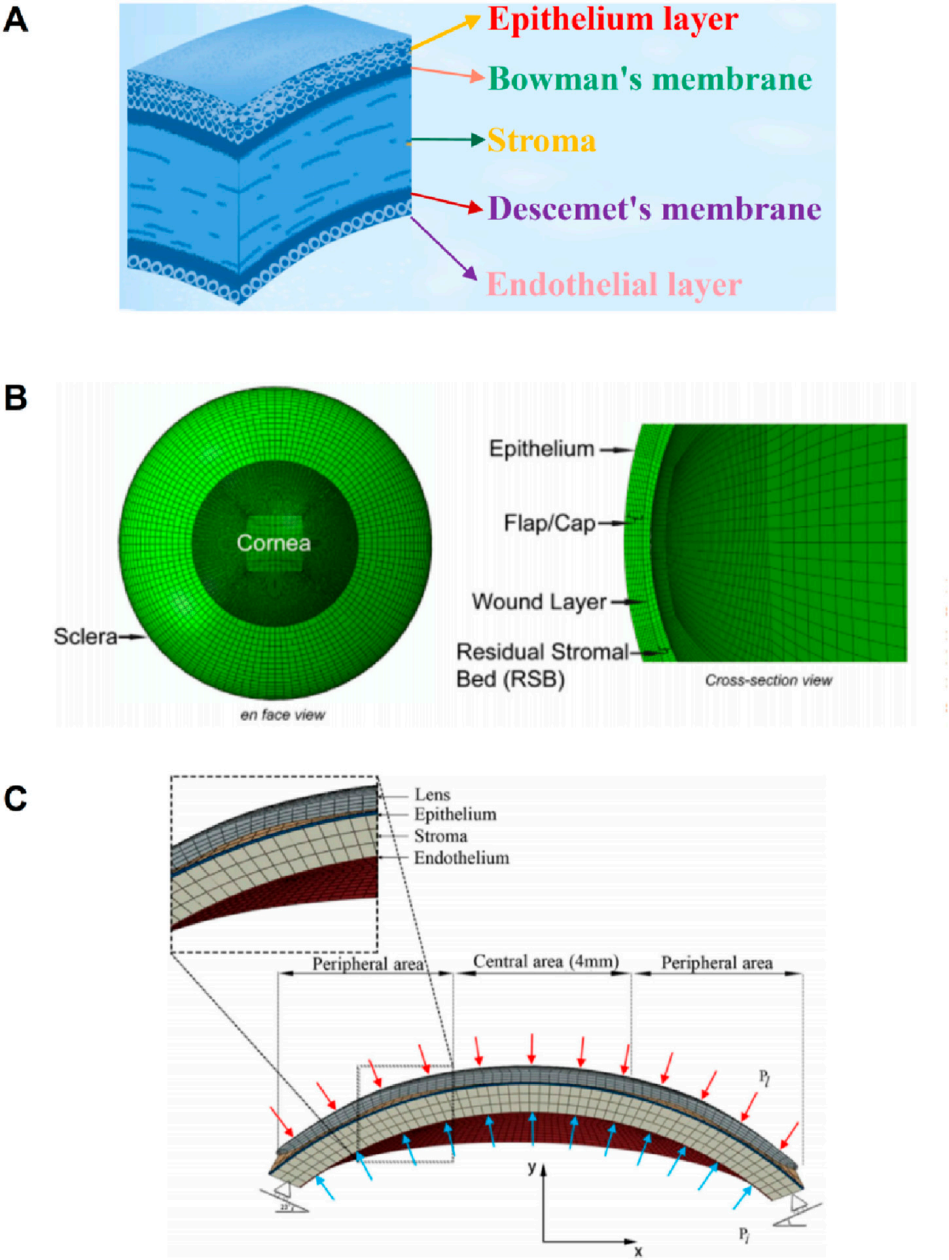
The diagram of corneal layering and layering cornea model (Wu et al., 2021; Roy and Dupps, 2011). **(A)** the schematic of corneal layering. **(B, C)** Layered corneal model.

The cornea’s absence of blood vessels, well-organized tissue, and near-dehydrated state all contribute to the cornea’s ability to repair itself and its transparency (Tuori et al., 1996). From the front to the back corneal tissue, there are five layers, namely, epithelial layer, bowman’s membrane, stromal layer, descemet’s membrane, and endothelial layer, as shown in Figure 6A (Parker et al., 2021). The epithelial layer consists of the cell layer and the basement membrane layer. The thickness of the epithelial layer is about 50 μm, the cell life is about 8–10 days, the cell regeneration ability is strong, and the repair after damage is faster. Limbal stem cells are found in the basal cell layer of the limbal, which plays an important role in the repair of the upper cortex. The bowman’s membrane has been formed in the embryonic stage, but the boundary between it and the stromal layer is not very clear. The boundary between it and the epithelial layer is obvious, about 10 μm, and it cannot be regenerated after injury. The stromal layer is the core of cornea structure, mechanics, and optics, with a thickness ranging from 400 μm to 550 μm. It is mainly composed of extracellular matrix such as collagen fibers and inorganic salts. There are about 300 stromal layers in the center of the human cornea and more than 500 layers in the corneal limbal. There are about 2900 endothelial cells per square millimeter in the endothelial layer, a total of more than 1 million endothelial cells, most of the cells are hexagonal, the older the number is less, because it is not mitotic, so it is not renewable. If the endothelial cells are more damaged or the number is reduced to a certain extent, it will cause corneal edema and other keratopathy. However, it is important to note that corneal delamination is technically difficult to achieve, and there is a lack of very accurate experimental data. The contact between layers is not clear, so there are few reports on corneal delamination in previous studies. For corneal refractive surgery, the surgical cutting site spans the epithelial layer, the bowman’s membrane, and the stroma layer, and the properties of each layer are often regarded as the same in the finite element simulation, which will also cause some calculation errors.

Larry et al. (2007) simulated corneal oxygen and corneal oxygenation by establishing 2-dimensional axisymmetric cornea and 3D contact lens, and analyzed oxygen distribution in different places of the cornea. The epithelium thickness is 0.050 mm; the stroma thickness is 0.475 mm; the endothelium thickness is 0.005 mm; the stroma perpherial thickness is 0.677 mm. Roy and Dupps (2011) established a 3D whole eyeball model in the normal eye to simulate LASIK. Since the cutting of the cornea in the LASIK surgery reaches the stromal layer, the model considers the epithelium, epithelium, wound layer, and residual stromal bed, as shown in Figure 6B. The effect of flap thickness and residual stroma on corneal surface stress distribution was analyzed with this model. Wu et al. (2021) divided the cornea into three layers: the epithelium, stroma, and endothelium. The layer thickness of the epithelial and Bowman’s membrane was presumed to be 50–57.5 μm, as was the Descemet’s membrane and endothelium thickness. An orthokeratology lens was applied to the epithelial layer to study the biomechanical response of the orthokeratology lens to the cornea, as shown in Figure 6C. Li et al. (2021) established a corneal model considering 5 layer structure and analyzed the effects of deep anterior lamellar keratoplasty and penetrating keratoplasty surgery on corneal biomechanical properties. The thickness of Descemet’s membrane and endothelium is 15 μm; the thickness of epithelial and Bowman’s membrane is 45 μm; the stroma thickness is 500 μm.

### 2.4 Boundary condition

In finite element calculation, the setting of boundary conditions also has a significant influence on the calculation results. For finite element models with only corneal tissue, the fixation of the boundary is usually at the corneal edge. On the one hand, the Angle setting of the corneal edge to the horizontal direction is an influencing factor. For example, in the model shown in Figure 2C, the corneal edges are parallel to the horizontal direction. In the model shown in Figure 4A, the corneal edge is parallel to the corneal cross-section at an Angle to the horizontal plane. In Figure 6C, the corneal margin is at an acute Angle to the horizontal surface. The shape of the corneal edge and the Angle from the horizontal plane are set differently by different researchers. However, the influence of corneal edge and horizontal Angle on simulation results has been rarely evaluated. On the other hand, the way the corneal edge is fixed also affects the calculation results. At present, the methods of fixing edge are also different in the literature reports. For example, Wang et al. (2022) and Wu et al. (2021) fixed the corneal edge by limiting the degree of freedom of angle at the corneal edge. Alastrue fixed the position of the corneal edge by limiting the degree of freedom of displacement. Qin et al. (2019) set the boundary conditions to limit all the degrees of displacement and rotation of the corneal edge. In short, the corneal edge shape and boundary conditions should be determined according to the calculation method and the actual research situation. For whole-eye models or models with more tissue components (such as Figure 5A), the setting of boundary conditions is more complicated, and the boundary conditions have a greater impact on the results. At this time, the boundary fixation method should be chosen carefully.

### 2.5 Analysis and discussion of geometric models

Very small changes in corneal shape can cause large changes in corneal refraction or performance. In the past, due to the limited technical level, researchers had a limited cognition level of cornea shape, and often simplified the cornea into a spherical or ellipsoid shape. After the model is simplified, the geometric model can be established directly by anatomic parameters without testing instruments. On the other hand, because the simplified models are more regular, it is easier to divide the grid in the finite element software, and the calculation is easier to converge, reducing the calculation cost. Corneal morphology varies greatly from patient to patient, for example, the difference in corneal thickness in the population can reach up to 300 μm. Conclusions calculated with a simplified model cannot be applied to everyone. Therefore, the idealized model can be regarded as a semi-quantitative or qualitative model to some extent. The advent of more and more sophisticated instruments has made it easy to accurately describe the geometry of the cornea. At present, a growing trend of clinical research is precision and individuation. Accurately describing the corneal morphology of specific patients greatly improves the accuracy of corneal research and simulation prediction results. In general, the accuracy of the 3D model is higher than that of the 2D model. Patient-specific models are more accurate than idealized models. It should be pointed out that, however, not all studies require patient-specific 3D models. This needs to be determined in conjunction with the needs and objectives of the research. In addition, the edge shape of the corneal model are also important factors affecting the calculation results. In the human eye, the corneal margin is connected to the sclera. The junction is often called the limbus, which interweaves corneal and scleral components. The thickness of the limbus is greater than that of the cornea. Moreover, the size, content, and interweaving direction of collagen fibers in the sclera are different from the distribution of the cornea collagen fibers. Therefore, the biomechanical properties and structural stiffness of the sclera are different from those of the cornea, and even differ greatly. Therefore, whether the shape of corneal limbus and material properties are considered in the model will affect the accuracy of the calculation results. However, there are few reports considering limbal geometry and material properties in current simulations.

There are few reports on the layered finite element model of cornea. There are two main reasons. One is the difficulty in calibrating the geometric parameters of each layer of the cornea. The difficulty of acquiring human cornea greatly limits the calibration of corneal parameters. Human corneas are often harvested from cadavers and stored in a solution. But inevitably, the cornea in the solution will absorb the solution leading to corneal swelling and distortion of corneal geometry. The thickness of each layer of the cornea is at the micron level, and the observation through the equipment may also cause large errors. Another reason is that the properties of corneal layers and the contact relationship between layers are difficult to describe accurately. The material properties and the number of cells in each layer of the cornea are different. It is technically difficult to separate the layers of the cornea, and there are currently no very effective means to fully peel the layers and to accurately determine the mechanical properties of the layers. Therefore, when the layered model is established, the material properties of each layer are set without an accurate basis. Theoretically, the contact relationship between the 5 layers is not the same. Whether there is a slip or friction relationship between the 5 layers remains to be studied. In brief, more accurate corneal stratification data needs further experiments and studies.

In finite element calculation, the geometry needs to be discrete into small elements. Usually, element is just an approximate representation of the actual structure geometry. Element type, shape and total number all affect the simulation results. In general, the more quantity of element, the more accurate the simulation results will be. But the problem is that the cost of computing increases. Therefore, it is necessary to analyze the convergence of the number of element in order to obtain more accurate results at the lowest computational cost. For the interest areas, the element should be partially encrypted for more accurate results. When meshing, hexahedral elements tend to give the best results at the lowest cost. In the field of computational mechanics, there is a lot of discussion and research on meshing. Hexahedral elements have a high degree of reliability and accuracy in the simulation of many incompressible biological materials (Karimi et al., 2021). In medical applications, Ramos and Simões (2006) reported that hexahedral mesh is more stable than tetrahedral mesh in femur model, and the result is less affected by mesh refinement. In the simulation of cardiac biomechanics, Oliveira and Sundnes (Lino De Oliveira and Sundnes, 2016) show that the secondary hexahedron is slightly superior to the secondary tetrahedron in mechanics. Benzley et al. (1995) reported that the computational stability and accuracy of linear hexahedral mesh are better than that of linear tetrahedral mesh. In the nonlinear elastic-plastic analysis experiment, the linear hexahedral mesh is slightly better than the quadratic tetrahedral mesh. Biswas and Strawn (1998) showed that for the same number of edges, a hexahedral mesh will produce a more accurate solution than a tetrahedral mesh. However, the most of patient-specific corneal and keratoconus shape is often irregular. It is difficult to fully divide into hexahedral element. It is also necessary to divide into tetrahedral element or wedge element. In some calculations these two elements are prone to “hourglassing phenomenon.” Therefore, these two types of elements should be used with caution and validity verification. In addition to the method of automatically generating mesh in finite element software, the hypermesh software is also an effective means of mesh division and processing. In some irregular models, hypermesh can effectively handle irregular regions and partition hexahedral meshes (Wang et al., 2017).

## 3 Corneal constitutive model

Fung (Fung, 1972) first pointed out that most biological soft tissues are viscoelastic materials, and so is corneal tissue. Corneal mechanical properties are more complex. The cornea contains both solid and liquid components. It is an anisotropic, viscoelastic, and incompressible material. Viscoelastic materials have both elastic and viscous deformation properties under the external forces (Williams, 1964; Sladek et al., 2022). The mechanical properties of viscoelastic materials are related to time, strain rate and other factors. The stress-strain relationship of corneal materials does not satisfy the linear elastic constitutive relationship. The elastic modulus of corneal materials is significantly different in the direction of longitude and latitude. In the finite element calculation, there are four common types of the corneal constitutive relations: linear elastic relation, exponential function relation, hyperelastic relation, and viscoelastic relation.

### 3.1 Linear elastic constitutive model

**FIGURE 7:**
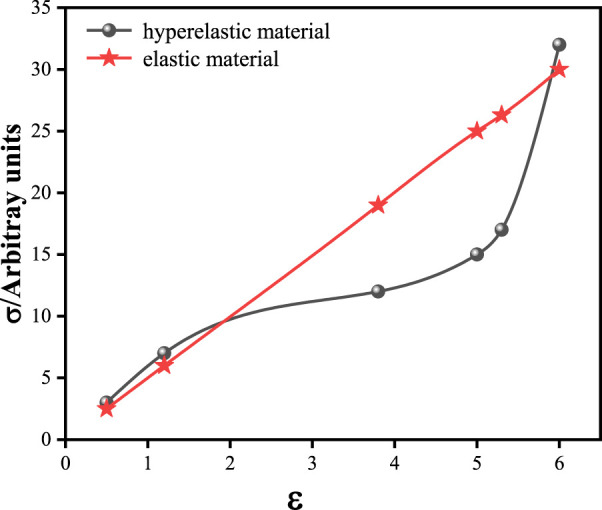
The stress-strain relationship diagram of hyperelastic and linear elastic materials.

The stress-strain relationship of the cornea is not linear. The observation of cornea *in vitro* showed that corneal fiber bundles had obvious directions. In the central cornea, the distribution of corneal collagen fibrolamella was generally cross-arranged. In the corneosclera margin, the distribution of collagen fibrolamella was circumferential. This distribution direction results in anisotropic mechanical properties of the cornea. Due to the difference in experimental conditions, experimental methods and materials, the measurement results of human corneal elastic modulus are wide. In order to simplify the calculation, many researchers treat the cornea as a linear elastic and isotropic material. The properties of corneal linear elastic materials can be determined by stretching experiments, and there are few undetermined parameters in the simulation. The only undetermined parameters are elastic modulus and Poisson’s ratio. Therefore, linear elastic properties are the most widely used in corneal simulation. The stress-strain relationship of linearly elastic materials is linear, as shown in Figure 7. The curve in Figure 7 is drawn from random data, not from some experiment or literature report. In this figure, the ordinate is stress in any unit, and the abscissa is strain. The red line describes the stress and strain relationship of the linearly elastic material, which is linear. The black lines describe the stress-strain relationship of the hyperelastic material, which is non-linear. Nguyen et al. (2019) simulated Corvis ST detection process by setting the cornea as linear elastic material. Bao et al. (2018) established a linear elastic three-dimensional corneal model, and simulated LASIK surgery to study the relationship between postoperative corneal eminence and various parameters. The cornea is regarded as a linear elastic material, which is easy to test in experiment and calculate in simulation. We summarized the values of elastic modulus reported in some literatures, as shown in Table 2. As can be seen from the table, the value of human eye elastic modulus reported in literature ranges from 0.05 MPa to 20.33 MPa. The values of corneal elastic modulus measured by different experimental methods are very different, and the range distribution of elastic modulus is relatively discrete. When different reference modulus values are set in finite element calculation, there will be large errors. Secondly, for the linear elastic model, the relationship between stress and strain changes linearly. The deformation and mechanical response of cornea are not linear. Especially when analyzing the surface or internal stress distribution of the cornea, the error caused by the linear elastic model may be more obvious.

**TABLE 2:** Elastic modulus and Poisson ratio parameters.

Elastic modulus	Poisson ratio	Ref.
0.363 MPa	0.49	Cavas et al. (2014)
14.3 MPa	0.45	Spevak and Babailov (2020)
1.5 MPa	0.49	Nguyen et al. (2019)
0.16–0.8 MPa	0.49	Fung (1972)
0.14–0.30 MPa	0.49	Qin et al. (2019)
0.40 MPa	0.42	Ramasubramanian et al. (2022)
20.33 MPa	0.49	Baek and Park (2019)
0.20 MPa	0.43	Bharathi et al. (2022)
0.05–19 MPa	0.49	Rossi et al. (2011)

### 3.2 Exponential function constitutive model

In order to avoid the error caused by linear elastic model describing corneal constitutive relation, the authors adopted the constitutive relation of exponential function to describe the nonlinear stress-strain relation of cornea. The function expression is shown in Formula 3:

$$\sigma =\alpha \left(e^{\beta \varepsilon }-1\right)$$ (3)

Where, *α* and *β* are material constants, *σ* and *ε* are stress and strain, respectively. The values of *α* and *β* in literature reports are shown in Table 3. For Poisson’s ratio, the cornea is generally considered to be almost incompressible row material. Therefore, 0.49 is set in both online elastic and exponential functions. But the flaws of the exponential model are also obvious. When the strain is small, the stress change is moderate. When the strain is a little greater, the stress will rise sharply. As reported by Asejczyk-Widlicka and Srodka (2020), when the stress variable is greater than 0.05, the stress value will increase sharply.

**TABLE 3:** Exponential function parameters.

α	β	Ref.
0.2 kPa	62	Asejczyk-Widlicka and Srodka (2020)
1.75 kPa	48.3	Fernandez et al. (2004) Xie et al. (2008)

### 3.3 Hyperelastic constitutive model

The stress-strain relationship of hyperelastic materials has several characteristics: hysteresis, stress relaxation, creep, Mullins effect or Pyane effect. The linear elastic constitutive method of human corneal material is not accurate enough to describe corneal hyperelastic properties. Fang et al. (2020a) established a post-LASIK corneal model containing a flap using a hyperelastic constitution. The biomechanical changes induced by the flap were evaluated and the displacement, stress and strain of the corneal surface were analyzed. Zhou et al. (2017) established a finite element model of the eyeball. The cornea and sclera were described by a self-defined nonlinear, incompressible, and anisotropic hyperelastic constitutive equation, and the particle swarm optimization algorithm was used to invert the constitutive parameters of the corneal material. Simonini et al. (2016) established a corneal model of patients after PRK, regarded cornea as anisotropic hyperelastic material. The influence of corneal cutting on corneal stress distribution and intraocular pressure fluctuation on refraction were analyzed. Fang Lihua et al. (2020) regarded cornea as heterogeneous, hyperelastic and isotropic material, and used Neo-Hookan constitutive model to study the mechanical parameters of acupuncture into the cornea. Elsheikh and Wang (2007) used the third-order Ogden hyperelastic model to describe the corneal constitutive, and used the iterative method to restore the state of the eye without intraocular pressure. Karimi et al. (2018) applied the Mooney-Rivlin constitutive to the conus and normal corneal models, studied the stress distribution of the two corneas. An artificial neural network algorithm for the diagnosis of keratoconus was proposed. Pandolfi et al. (Pandolfi and Manganiello, 2006; Pandolfi et al., 2009) established a hyperelastic three-dimensional finite element corneal model considering the distribution of collagen fibers. He believed that the distribution of stress and refractive power on the corneal surface and in the direction of corneal thickness were related to material properties and fiber distribution. This study did not consider the differences in the distribution of collagen fibers in different corneas and the differences in mechanical parameters.

In the present study of corneal finite element simulation, the cornea as a hyperelastic material is closer to the real cornea properties. Common hyperelastic models include Mooney-Rivlin model, Neo-Hookean model, Yeoh model, Ogden model, and Holzapfel-Gasser-Ogden (HGO) models (Peng et al., 2021; Horgan, 2021; Melly et al., 2022; Lohr et al., 2022; Anssari-Benam et al., 2022; Karlsson et al., 2023; Gruber et al., 2024). The correspondence between the stress tensor and strain tensor of hyperelastic materials is not linear and does not satisfy Hooke’s law, but corresponds to in elastic potential energy function, as shown in Figure 7. The constitutive relation can be given by strain energy function. The characteristics of hyperelastic materials are that the stress is only related to the strain, and has nothing to do with the path, rate, and history of the load applied. The deformation of the material can be restored after the load is released. The expression of strain energy function is shown in Table 4. These hyperelastic constitutive models are widely used in cornea research. The Mooney-Rivlin form is seen as an extension of the Neo-Hookean form, where one term is determined by the second invariant of the isotonic Cauchy-Green tensor. In many cases, the Neo-Hookean form is closer to the experimental data than the Mooney-Rivlin form. The accuracy of the two models is similar. Their strain energy is a linear function of invariants, and they cannot reflect the ‘steep rise’ behavior of the stress-strain curve in the large strain part, but they can well simulate the characteristics of materials under small and medium strains. For Ogden model, the strain energy is variable by three principal elongation ratios: $\lambda_{1}$ , $\lambda_{2}$ , $\lambda_{3}$ . If N = 1, $\alpha_{1}=2$ , $\alpha_{2}=-2$ , then get the Mooney-Rivlin model. If N = 1, $\alpha_{1}=2$ , then get the Neo-Hookean model. In the Ogden model, $\mu_{0}$ is all determined by coefficient: $\mu_{0}=\sum_{i=1}^{N}\mu_{i}$ . Arruda-Boyce model and Van-der-Waals model are also common hyperelastic models, but these two constitutive relations are obtained based on thermodynamic statistical methods (Kumar et al., 2022; Castellanos-Gomez et al., 2022). Thermodynamic coupling is rarely involved in corneal simulation, so these two models are not described and discussed in this review. Where, ${\bar{I}}_{1}=\sum_{i=1}^{3}{{\bar{\lambda }}_{i}}^{2}$ , ${\bar{I}}_{2}=\sum_{i=1}^{3}{{\bar{\lambda }}_{i}}^{\left(-2\right)}$ ; $\bar{E_{\alpha }}=\kappa \left({\bar{I}}_{1}-3\right)+\left(1-3\kappa \right)\left({\bar{I}}_{4\left(\alpha \right)}-1\right)$ ; ${\bar{I}}_{4\left(\alpha \right)}=A_{\alpha }\bar{C}A_{\alpha }$ ; $\bar{C}=J^{-2/3}C$ ; $J={\left(\det C\right)}^{1/2}$ ; $C=F^{T}F$ ; $C_{\text{ij}}$ , $D_{i}$ , α , and *μ* are material constants; ${\bar{I}}_{i}$ is the first invariant of the modified right Cauchy-Green tensor; ${\bar{\lambda }}_{i}$ is deviatoric principal stretches; *J* is the elastic volume ratio; $C_{10}$ is matrix stiffness; $\frac{1}{D}\left(\frac{J-1}{2}-\ln J\right)$ is corneal volume strain energy; *D* represents the inverse of the volumetric modulus. Considering that the cornea is almost incompressible material, it is set to a very small value in the setting. *k*_1_ represents fiber stiffness; *n* is the number of families of fibers; *k*_2_ is a dimensionless parameter referring to fiber nonlinearity; ${\bar{E}}_{\alpha }$ characterizes the deformation of the family of fibers with mean direction; κ describes the level of dispersion in the fiber, 0≤κ≤1/3 ; κ=0 indicates that collagen fibers are strictly distributed in the main direction of collagen fibers; κ=1/3 indicates that collagen fibers are randomly distributed. *A*_α_ is the direction vector of the fiber bundle; ${\bar{I}}_{4\left(\alpha \right)}$ represents the square of the stretch along the direction of the α-th family of fibers; *F* is the deformation gradient; *C* is right Cauchy-Green strain tensor. The parameter settings of these models from literature reports are shown in Table 5.

**TABLE 4:** Strain energy function of hyperelastic model.

Model	Strain energy function
Mooney-Rivlin	$U=C_{10}\left({\bar{I}}_{1}-3\right)+C_{01}\left({\bar{I}}_{2}-3\right)+\frac{1}{D_{1}}{\left(J-1\right)}^{2}$
Neo-Hookean	$U=C_{10}\left({\bar{I}}_{1}-3\right)+\frac{1}{D_{1}}{\left(J-1\right)}^{2}$
Ogden	$U=\sum_{i=1}^{N}\frac{2\mu_{i}}{\alpha_{i}^{2}}\left({\bar{\lambda }}_{1}^{\alpha_{i}}+{\bar{\lambda }}_{2}^{\alpha_{i}}+{\bar{\lambda }}_{3}^{\alpha_{i}}-3\right)+\sum_{i=1}^{N}\frac{1}{D_{i}}{\left(J-1\right)}^{2i}$
Yeoh	$U=\sum_{i=1}^{N}C_{i0}{\left({\bar{I}}_{1}-3\right)}^{i}+\sum_{i=1}^{N}\frac{1}{D_{i}}{\left(J-1\right)}^{2i}$
HGO	$U=C_{10}\left({\bar{I}}_{1}-3\right)+\frac{1}{D}\left(\frac{J^{2}-1}{2}-\ln J\right)+\frac{k_{1}}{k_{2}}\sum_{\alpha =1}^{N}\left(\left(e^{k_{2}{\bar{E_{\alpha }}}^{2}}-1\right)\right)$

**TABLE 5:** Hyperelastic model parameters.

*Model*	*Values*		Ref.
Mooney-Rivlin	Material-1	*C*_10_ = 0.3MPa, *C*_01_ = 0.12 MPa	Bekesi et al. (2016)
	Material-2	*C*_10_ = 0.55MPa, *C*_01_ = 0.17 MPa	
	Material-3	*C*_10_ = 0.62MPa, *C*_01_ = 0.41 MPa	
Neo-Hookean	*D* = 0, *C*_10_ = 0.15–1.00 MPa		Han and Yang (2019)
Ogden	First-order	*μ*_1_ = 0.0541 MPa *α*_1_ = 110.4	Bao et al. (2018)
	First-order	*μ*_1_ = 0.6042 MPa *α*_1_ = 16.45	Fang et al. (2020b)
	Second-order	*μ*_1_ = 0.0260 MPa *α*_1_ = 75.11 *μ*_2_ = 0.0229 MPa *α*_2_ = 63.70	Lago et al. (2015a)
	Second-order	*μ*_1_ = 0.0035 MPa *α*_1_ = 104.06 *μ*_2_ = 0.0035 MPa *α*_2_ = 103.94	Song et al. (2021)
Yeoh	Third-order	*D* = 0, *C*_10_ = 0.81 MPa,*C*_20_ = 56.05 MPa,*C*_30_ = 2332.26 MPa	Ariza-Gracia et al. (2016)
HGO	*C*_10_ = 0.050MPa, *D* = 0, *k*_1_ = 25–130.9MPa, *k*_2_ = 2490, *κ* = 0.33329		Ariza-Gracia et al. (2016)
	*C*_10_ = 0.025MPa, *D* = 0, *k*_1_ = 0.092MPa, *k*_2_ = 785.68, *κ* = 0.3		Xu et al. (2018)
	*C*_10_ = 0.220MPa, *D* = 0, *k*_1_ = 0.615MPa, *k*_2_ = 121.633, *κ* = 0.3		Xiang et al. (2018)

The determination of material parameters is the result of experimental fitting by researchers. However, it can be seen from the literature that the parameters used by different researchers for the same constitutive model are not the same, or even quite different. This is because there are many factors that affect the results of corneal hyperelastic parameters, such as the difference of materials, the difference of mechanical testing methods, and different fitting algorithms. As for the sampling factors, the common corneal sources are cadavers and corneal surgical scraps. First of all, corneal cells and collagen fibers of different ages have great differences, and their macroscopic mechanical properties are also different. The method of corneal preservation and the time after cornea isolation also have a great influence. Next is the area where the cornea is surgically obtained. The remaining corneal tissue after corneal transplantation is often the corneosclera region, which is thicker than the central cornea. The corneas obtained from SMILE surgery tend to be the central cornea. The anisotropy difference of cornea also directly leads to the different mechanical properties of different regions. For the mechanical testing methods, the common methods include axial tensile test and expansion test. The stretching test is to cut the cornea into strips for stretching test, while the swelling test is generally to test the cornea as a whole. Two different test methods are the cause of the difference in parameters. Since the hyperelastic constitutive model needs many undetermined parameters in the calculation software, it is necessary to use the calculation software to fit the measured stress-strain curve. For a certain test result, different fitting algorithms may fit many kinds of parameter combination results that are consistent with the experimental curve. Xiang et al. (2018) conducted a uniaxial stretching experiment with the corneal lens removed by SMILE surgery. Parameters of HGO model were fitted by least square method to 34 experimental results. The average values of *C*_10_, *k*_1_ and *k*_2_ were 0.220, 0.615 and 121.633. These values are significantly different from the results of Ariza-Gracia et al. (2016) fitting (*C*_10_ = 0.05, *k*_1_ = 130.9, *k*_2_ = 2490). Xiang et al. (2018) also suggested that the change of κ value has a very large effect on corneal stress-strain, and also affects the values of *k*_1_ and *k*_2_. The κ value of each cornea is different, which means the results of other parameters fitting may not be accurate.

### 3.4 Viscoelastic constitutive model

**FIGURE 8:**
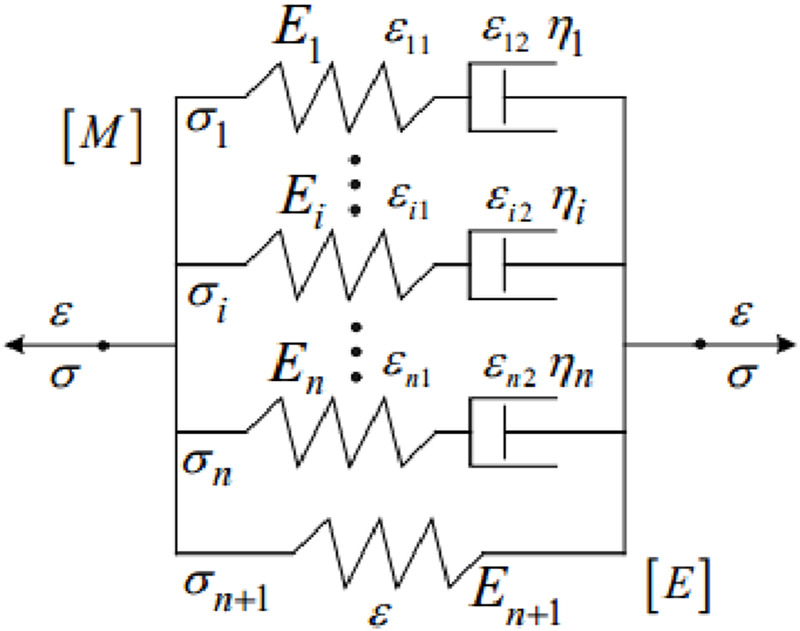
Modified Maxwell viscoelastic model of cornea (Melly et al., 2022).

Compared with linear elastic materials and hyperelastic materials, viscoelastic materials are more accurate in describing the constitutive relationship of the cornea. However, viscoelastic constitutive complexity is higher than linear elastic and hyperelastic constitutive complexity. Therefore, there are few corneal viscoelastic constitutive models reported in the literature. Kling et al. (2014) developed a viscoelastic finite element model to predict the experimental deformation response of the cornea to inflation under different conditions. Corneal tissue was studied by linear viscoelastic constitutive method. Therefore, only the shear response is considered, as it usually dominates the volume response. The viscoelastic constitutive is shown in Formula 4:

$$\sigma =\int_{0}^{t}2G\left(t‐\tau \right)\frac{\text{de}}{d\tau }\text{dt},G\left(t\right)=G_{0}\cdot \left[\alpha_{1}^{G}\cdot \exp\left(‐\frac{t}{\tau_{1}^{G}}\right)\right],G_{0}=G_{\infty }+G_{1},\alpha_{1}^{G}=\frac{G_{i}}{G_{0}}$$ (4)

where σ is the Cauchy stress, e the deviatoric strain and τ past time. G(t) is the Prony shear modulus; $\alpha_{1}^{G}$ is the relative modulus; $G_{0}$ is instantaneous elastic moduli; $G_{\infty }$ is infinite elastic moduli; $G_{1}$ is the current shear elastic moduli. $\tau_{1}^{G}$ is the relaxation times for the Prony component. Many viscoelastic models are composed of elastic and viscous elements (Banks et al., 2011; Bonet, 2001). MaxwelL model and Kelvin model are the basic models of viscoelasticity. Maxwell model is a series connection between spring and shock absorber (Renaud et al., 2011). The Kelvin model is a parallel connection between the spring and the shock absorber (Xu et al., 2015). The relationship of stress-strain is shown in Formula 5:

$$\sigma \left(t\right)=E_{equ}\cdot \varepsilon \left(t\right)+\sum_{i=1}^{m}E_{i}\cdot \overset{•}{\varepsilon }\cdot \tau_{i}\cdot \left(1-e^{-\frac{t}{\tau_{i}}}\right)$$ (5)

where Eequ is the equilibrium modulus, Ei is the relaxation modulus of the *i*th branch, $\tau_{i}$ is the time constant of the *i*th branch, ε is the strain, and $\overset{•}{\varepsilon }$ is the strain rate. Su et al. (2015) proposed a modified Maxwell viscoelastic model of the cornea from the perspective of material mechanics, as shown in Figure 8. The viscoelastic parameters were determined by the uniaxial tensile test and stress relaxation test. Through the simulation of corneal material properties, the validity of the superviscoelastic model is verified. The model is composed of Maxwell model [*M*] in parallel. Each Maxwell model [*M*] has a series damper. If the external load is applied long enough, the internal stress of the material will be close to zero. In Figure 8, the elastic element is connected in parallel with the generalized Maxwell model. This model is also called the modified Maxwell viscoelastic model of the cornea. The solid properties shown by the parallel elastic element [*E*] reflect the residual stress of the cornea in the relaxation curve. The stress and strain in the model are described by the Formula 6:

$$\sigma =\sum_{i=1}^{n+1}\sigma_{i}\;\varepsilon =\varepsilon_{i1}+\varepsilon_{i2}\;\left(i=1,2,\ldots i,\ldots ,n\right)$$ (6)

where *σ*_*i*_ is the stress in the *i*th sub-model; *ε*_*i*1_ is the strain of the *i*th elastic element; *ε*_*i*2_ is the strain of the *i*th viscous element.The constitutive equation of [M] model can be expressed as Formula 7:

$$\sum_{i=1}^{n}\sigma_{i}+\sum_{i=1}^{n}\frac{\eta_{i}}{E_{i}}\sigma_{i}=\sum_{i-1}^{n}\eta_{i}\varepsilon_{i}$$ (7)

*E*_*i*_ and *η*_*i*_ are the elastic modulus and viscosity coefficient of the *i*th Maxwell model element.

### 3.5 Comparison and analysis of different constitutive models

An ideal constitutive model should have the following requirements: contain as few parameters as possible; it can correctly predict the arbitrary complex deformation behavior of materials. But there seems to be some contradiction between these two points. In general, the higher the order of a constitutive equation, or the more undetermined parameters, the higher the accuracy of the constitutive equation. However, the more parameters to be determined, the more difficult the calculation is. The appropriate constitutive relation should be selected according to the different simulation and experiment requirements. The models in Table 5 have different characteristics. Yeoh model and Neo-Hookean model are the simplest in terms of form. Yeoh model requires few undetermined parameters, which can be obtained only from uniaxial tensile data. The model has a wide range to describe the superelastic behavior and deformation of materials. It is also most commonly used by researchers in corneal simulations. However, when the stress-strain curve of equiaxial tensile is predicted, the error is large and it is easy to show the phenomenon of “soft phenomenon.” Therefore, when the corneal experiment is through uniaxial stretching experiment, it is more suitable to use this model to fit the parameters. When dealing with complex strain states with large deformation, such as corneal biaxial tensile tests, large deviations can occur. In this case, the model should be used with caution. The Neo-Hookean model is derived based on classical statistical thermodynamics. The model is simple in form, requires only two parameters, and has strong universality. It is generally suitable for small deformation uniaxial drawing with strain rate of 30%–40%. Since corneal are generally considered incompressible material, the number of undetermined parameters in the model is reduced to one. Only one parameter, the initial shear modulus, needs to be fitted from the experimental data, and the amount of testing is small. The polynomial model proposed by Mooney-Rivlin can accurately simulate the stress-strain relationship under various deformation modes. However, the model is complex and requires several experiments to obtain individual parameters. The model has 3 orders, 5 orders, and 9 orders. The higher the order, the higher the accuracy of calculation. With the increase of the order, it becomes more difficult to determine the material parameters. Ogden model can also accurately simulate stress-strain relationship under various deformation modes, but it also needs to obtain parameters through multiple experiments. The HGO hyperelastic model takes into account the distribution direction of stromal collagen fiber clusters in human cornea, which can more accurately describe the biomechanical properties of cornea *in vitro* and *in vivo*. Moreover, there are also fewer undetermined parameters. In the simulation of human cornea, it is the most popular and widely used constitutive model.

Theoretically, the accuracy of viscoelastic model is higher than that of hyperelastic model. However, it can also be seen from the form that there are three mathematical variables of stress-strain-time in the viscoelastic model, which is more complicated than the hyperelastic model. The viscoelastic model is mainly composed of elastic elements and viscous elements in parallel or in series. The model can be composed of two or more components. The more the number of components, the more complex the connection mode, the more difficult the parameters to be determined. But each model has some drawbacks. For example, the Maxwell model consisting of an elastic element and a viscous element in series cannot describe the phenomenon of stress relaxation. The Kelvin model, which consists of an elastic element and a viscous element in parallel, cannot describe the instantaneous elastic phenomenon or the plastic deformation. Other viscoelastic models (Ering, Burgers, Bingham, Nishihara) also have mechanical states that cannot be described exactly. Although the more complex the model, the higher the accuracy of the description. In selecting the surface model, we should choose the appropriate viscoelastic model to calculate according to different research problems.

For different simulation case, the appropriate constitutive model should be selected for simulation. The choice of constitutive model should not blindly pursue complexity. For example, Wu et al. (2021) studied the corneal biomechanical response of orthokeratology. The stress changes of corneal center and peripheral region were analyzed for models with different corneal curvature and corneal thickness under the action of orthokeratology. This kind of model focuses more on qualitative analysis than on the absolute value of stress or strain at a certain location. In this case, we can appropriately choose a simpler model, such as the first or second-order Ogden model. However, for the simulation analysis of myopia surgery with corneal flap or cap, we often pay more attention to the numerical value of the local area. For example, the amount of displacement and stress in the corneal cap or flap area. In this case, we choose as complex constitutive models as possible for simulation, such as HGO model or viscoelastic model.

The human corneathe environment is usually room temperature or constant temperature. Therefore, no matter what kind of model, the influence of temperature on constitutive relationship is often ignored. If the simulated case is not constant temperature, the effect of temperature on the material properties should be considered in the constitutive relationship.

## 4 Prospect and outlook

The development of finite element software has played an important role in promoting the study of cornea. The finite element model is often used in the study of eye diseases such as prediction of corneal refractive surgery, early prediction and diagnosis of keratoconus, diagnosis and treatment of eye trauma and glaucoma. Over the past few decades, researchers have established finite element models for different purposes and methods to study the human cornea, which has greatly promoted research in cornea-related fields. It plays an important role in our further understanding of corneal material properties and biomechanical response, and helps us in-depth analysis of postoperative corneal and keratopathy research. Moreover, virtual surgical simulation system using computer simulation is a new development direction in the future. Compared with traditional surgery, virtual surgery has the advantages of non-injury, repeatability and referencability. It saves the cost and time of training medical personnel and greatly reduces the risk of operation by unskilled personnel, which has special significance for improving the efficiency and quality of medical education and training and improving the unbalanced development of medical operation level. Finite element model is the most important part of virtual surgical system. Therefore, the accurate establishment of the corneal finite element model lays an important foundation for exploring the research of cornea-related surgical digital system.

The accuracy of geometric description of cornea plays an important role in visual simulation results. The tissue around the cornea and its morphology also affect the corneal simulation results. Like the effects of aqueous humor, the effects of the pericorneal sclera. Aqueous humor occupies a small volume, it is believed that the influence on the cornea is small. However, the sclera plays the role of boundary conditions in simulation, and the influence of boundary conditions on simulation results cannot be ignored. It is difficult to accurately describe the geometry of cornea and surrounding tissues with a single instrument. We can combine multiple devices, using the advantages of each device to try to achieve an accurate description of the cornea and surrounding tissues. For the surface of the cornea, the Pentacam can be used to obtain accurate information about the front and back surfaces of the cornea. For peripheral tissue, CT and MRI techniques can effectively obtain geometric information. The combined application of multiple devices may lead to a more comprehensive and accurate description of the geometry of the cornea and surrounding tissues. In addition, we obtain the cornea *in vivo* as a state of intraocular pressure. This state is not the same as the state before applying intraocular pressure in the simulation. Therefore, before the calculation, it is necessary to find the shape of the cornea in the absence of intraocular pressure. Ariza-Gracia et al. (2016) quantitatively analyzed the calculation results of zero-pressure model and image-based model. It is proved that the calculation results of the two models are significantly different. Therefore, zero-pressure state is also an important factor to be considered in geometric models.

Characterization of corneal material properties is also an important challenge. Because of individual differences, corneal biomechanical properties are different in different populations, different ages, and after corneal injury. However, at present, there are few relevant studies, which also leads to the error of corneal parameter setting during simulation. Therefore, the constitutive relationship of cornea in different populations remains to be studied. It is still an important way to obtain corneal constitutive relationship through corneal experiment. The stress-strain relationship of cornea can obtain by corneal swelling test, creep test, stress relaxation test and other mechanical properties tests. It may be more accurate to deduce the parameters of the constitutive relation from the stress-strain relation. It is difficult for some stress-strain data to fit well into the existing models in finite element software. We can add subroutines to the constitutive model so that the experimental data can be better fitted to the constitutive model.

Ultimately, this review aims to provide some thoughts of human cornea finite element model of geometry model and constitutive model for related researchers, and we hope will promote their future research.
